# Proteomics Reveals that Methylmalonyl-CoA Mutase Modulates Cell Architecture and Increases Susceptibility to Stress

**DOI:** 10.3390/ijms21144998

**Published:** 2020-07-15

**Authors:** Michele Costanzo, Marianna Caterino, Armando Cevenini, Vincent Jung, Cerina Chhuon, Joanna Lipecka, Roberta Fedele, Ida Chiara Guerrera, Margherita Ruoppolo

**Affiliations:** 1 Department of Molecular Medicine and Medical Biotechnology, School of Medicine, University of Naples Federico II, 80131 Naples, Italy; michele.costanzo@unina.it (M.C.); marianna.caterino@unina.it (M.C.); armando.cevenini@unina.it (A.C.); 2 CEINGE—Biotecnologie Avanzate s.c.ar.l., 80145 Naples, Italy; fedeler@ceinge.unina.it; 3 Proteomics Platform Necker, Université de Paris—Structure Fédérative de Recherche Necker, Inserm US24/CNRS UMS3633, 75015 Paris, France; vincent.jung@inserm.fr (V.J.); cerina.chhuon@inserm.fr (C.C.); joanna.lipecka@inserm.fr (J.L.)

**Keywords:** proteomics, label-free quantification, methylmalonyl-CoA mutase, MUT, methylmalonic acidemia, CRISPR/Cas9, bioinformatics, pathway analysis, ROS, inborn error of metabolism

## Abstract

Methylmalonic acidemia (MMA) is a rare inborn error of metabolism caused by deficiency of the methylmalonyl-CoA mutase (MUT) enzyme. Downstream MUT deficiency, methylmalonic acid accumulates together with toxic metabolites from propionyl-CoA and other compounds upstream of the block in the enzyme pathway. The presentation is with life-threatening acidosis, respiratory distress, brain disturbance, hyperammonemia, and ketosis. Survivors develop poorly understood multi-organ damage, notably to the brain and kidneys. The HEK 293 cell line was engineered by CRISPR/Cas9 technology to knock out the *MUT* gene (MUT-KO). Shotgun label-free quantitative proteomics and bioinformatics analyses revealed potential damaging biological processes in MUT-deficient cells. MUT-KO induced alteration of cellular architecture and morphology, and ROS overproduction. We found the alteration of proteins involved in cytoskeleton and cell adhesion organization, cell trafficking, mitochondrial, and oxidative processes, as validated by the regulation of VIM, EXT2, SDC2, FN1, GLUL, and CHD1. Additionally, a cell model of MUT-rescuing was developed in order to control the specificity of MUT-KO effects. Globally, the proteomic landscape of MUT-KO suggests the cell model to have an increased susceptibility to propionate- and H_2_O_2_-induced stress through an impairment of the mitochondrial functionality and unbalances in the oxidation-reduction processes.

## 1. Introduction

Methylmalonic acidemias (MMAs) consist of a group of autosomal recessive inherited metabolic disorders whose pathogenesis involves defects in the catabolism of propionyl-CoA. The propionyl-CoA produced from the degradation of cholesterol, branched-chain amino acids (valine, isoleucine, methionine, threonine), and the β-oxidation of odd-chain fatty acids is converted into methylmalonyl-CoA, which is further converted into succinyl-CoA, required for energy production in the Krebs cycle [1]. In the mitochondria of MMA patients, the conversion of methylmalonyl-CoA into succinyl-CoA, catalyzed by the vitamin B12-dependent methylmalonyl-CoA mutase (MUT) enzyme, is altered. This can be due to: (i) Deficiency of MUT apoenzyme or (ii) defect in the synthesis or transport of adenosylcobalamin (MUT cofactor). In the first case, the disease is referred to as isolated MMA, indicated by mut^0^ or mut^−^ phenotypes if the deficiency is total or partial, respectively [1]. On the other hand, alterations in the cobalamin (Cbl) metabolism may cause the so-called Cbl-related MMA. In particular, eight complementation groups (*cblA-cblH*) of enzymes are known to be involved in the processing of Cbl, from dietary transport until the formation of cofactors, and mutations in each of these genes are associated with a specific disorder [2]. The blockage of the enzymatic reaction catalyzed by MUT (either for MUT or Cbl defects) leads to an increase in the levels of methylmalonic acid, a hallmark of this group of diseases [3]. There are secondary changes in acylcarnitines, amino acids, and organic acids. These metabolites are easily detectable and quantifiable by metabolomics approaches, using liquid chromatography—tandem mass spectrometry (LC-MS/MS) or gas chromatography—mass spectrometry (GC-MS), in dried blood spots or urine or plasma from patients to perform diagnosis [4,5]. Due to the development of very sensitive technologies and appropriate second-tier tests, which allow discriminating between disorders of the cobalamin metabolism, MMA is included in the panel of diseases for the expanded newborn screening in several countries, including Italy [6].

Our work aims at understanding the mechanisms of cellular damage in the MMA disease. The majority of MMA complications arise from brain damage, on which neurological alterations and movement disorders depend. Acute brain alterations are promoted by concurring hyperammonemia, acidosis, brain edema, and hypoperfusion [7], causing permanent learning and developmental disturbances. When the brain damage becomes chronic, this affects the basal ganglia particularly, leading to movement and tone alterations [7,8]. Nonetheless, the mechanisms underlying the brain damage are still unknown. In addition, the renal function is commonly compromised in MMA patients, even the pathogenesis of kidney injury is not clear. Renal insufficiency and chronic kidney disease are partially resolved after kidney transplantation [1,8]. Actually, general organ metabolic alterations are thought to be caused by the breakdown of branched-chain amino acids that lead to the accumulation of circulating toxic acidic metabolites. Thus, metabolic acidosis induces a drop in bicarbonate levels. Lactic acidosis and hyperammonemia were shown to be important metabolic alterations of MMA [7,8]. Sadly, no efficient therapy exists for MMA, thus a dietary restriction of propiogenic amino acids is mandatory to reduce the substrate of deficient MUT, concomitant to supplementation with L-carnitine and vitamins [9,10]. Although useful in the clinical management of patients, these therapeutic strategies do not prevent the long-term brain and renal abnormalities.

Results of a previous work on a MUT-knockdown neuroblastoma cell model [11] suggested that the transient silencing of *MUT* gene was not sufficient to input long-term decompensation due to the absence of the protein. For this reason, we have developed a new cellular model for isolated MMA by stably knocking out the *MUT* gene in the HEK 293 cell line using CRISPR/Cas9 genome editing technology. We performed a global proteomic analysis to describe protein changes strictly connected to MUT absence and related altered pathways. Altogether, the results obtained shed new light on the molecular mechanisms of cellular damage, including alterations of cell architecture and morphology in combination with the acquisition of a higher sensitivity to stress.

## 2. Results

### 2.1. CRISPR/Cas9-Mediated MUT Gene Knockout in a HEK 293 Cell Line

**Figure 1:**
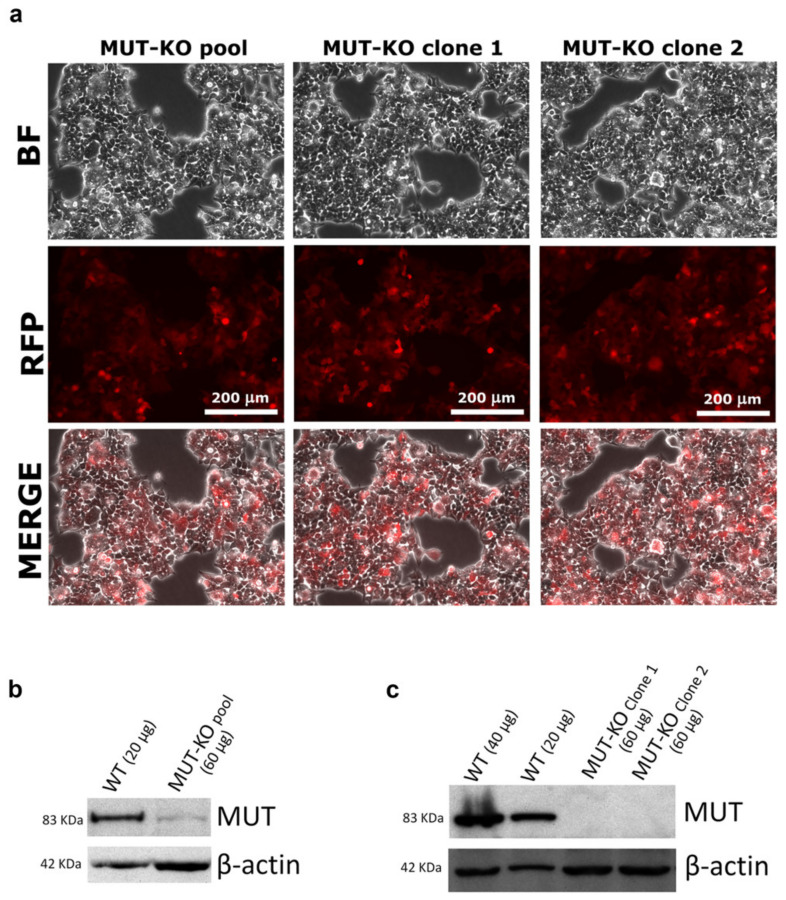
Analysis of HEK 293 cells after genome editing and culturing in a selective medium for methylmalonyl-CoA mutase knockout (MUT-KO). (**a**) Microscopy images of CRISPR/Cas9-modified cells. After transfection, cells were observed with a 20× objective and images were acquired with the Leica LAS AF software. MUT-KO pool: Whole CRISPR/Cas9-transfected cell population after selection with puromycin. MUT-KO clones: Cell populations isolated from single progenitor cells within the MUT-KO pool. RFP: Fluorescence signal from the red fluorescent protein detected with a Leica N3 filter cube. BF: Phase-contrast bright field. The Western blot (WB) analysis of MUT levels in the (**b**) MUT-KO pool and (**c**) two single cell clones (namely, MUT-KO clone 1 and 2), isolated from the MUT-KO pool. In both WBs, wild type (WT) cells were used as a control of MUT expression; β-actin was used as the loading control.

In order to establish a cell line knocked out for the *MUT* gene, the HEK 293 cells genome was manipulated using a CRISPR/Cas9 technology. Targeting the *MUT* gene, the vectors mediated the insertion of a construct able to express a red fluorescent protein (RFP) and a gene conferring puromycin resistance. After culturing in an antibiotic-selective medium, the cells still adherent showed red fluorescence (MUT-KO pool, Figure 1a), hence indicating that the homology-directed repair process (following a Cas9-mediated DNA cut) happened with high efficiency. After seven days, the MUT-KO pool still retained MUT protein expression, even if at a very low level (Figure 1b). In the following weeks, the pool of puromycin-resistant cells was properly diluted and plated, in order to have separate colonies each formed by a single resistant cell clone. The RFP signal was also used as a marker for the selection of clones. The first two clones (namely, MUT-KO clone 1 and clone 2) analyzed by WB (Figure 1c) showed the complete absence of MUT expression and they still retained red fluorescence (Figure 1a). Clone 2 was chosen to be used for the following experiments showing no significant expression of MUT mRNA by qRT-PCR (Supplementary Figure S1). Hereinafter, clone 2 will be simply indicated as MUT-KO.

### 2.2. Methylmalonic Acid and Propionylcarnitine Are Increased in MUT-KO Cells

**Figure 2:**
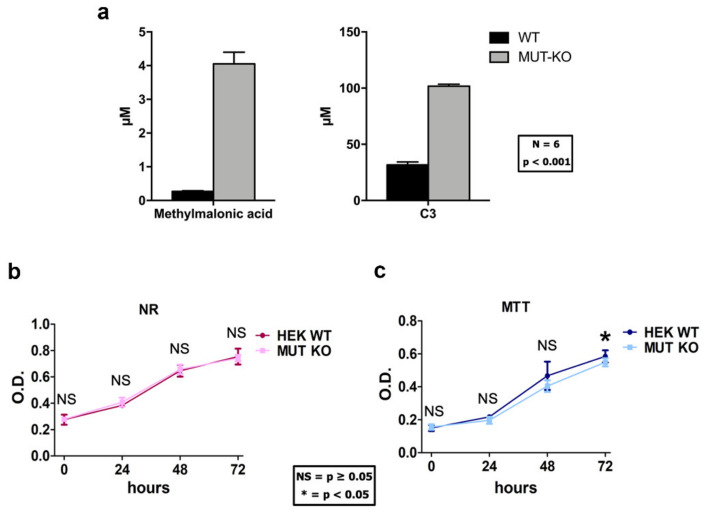
Measurement of methylmalonic acidemia (MMA) metabolites biomarkers and growth curves in HEK 293 cells after genome modification. (**a**) The levels of methylmalonic acid and propionylcarnitine (reported as C3) were measured by targeted LC-MS/MS. For both metabolites, significantly higher levels were detected in MUT-KO cells in comparison with WT ones. Concentrations were normalized to the protein content of each sample and reported as µM. For each condition, six biological replicates were employed. Data are reported as mean ± standard error of the mean (SEM); a one-way two-tail *t*-test *p*-value for both comparisons is ≤0.001. WT and MUT-KO cells were analyzed by (**b**) Neutral-red (NR) and (**c**) MTT assays. Data are reported as mean values of three independent experimental replicates and error bars represent the standard deviation (SD). A one-way two-tail paired *t*-test was used to calculate the statistical significance of differences between the tested cell types. OD: Optical density; *: *p* < 0.05; NS: Not significant (*p* > 0.05).

In the mitochondria of MMA patients, when methylmalonyl-CoA mutase is not present or has a defective activity, increased levels of methylmalonyl-CoA activate methymalonyl-CoA hydrolase enzyme, which removes the CoA group from the molecule producing methylmalonic acid. In addition, also propionyl-CoA accumulates and conjugates to free carnitine producing propionylcarnitine (C3). Methylmalonic acid and C3 are, in fact, biomarkers for the early diagnosis of MMA in the newborn screening program for inherited metabolic diseases [4,6]. Hence, the validity of our cell model was confirmed by targeted LC-MS/MS by increased levels of methylmalonic acid and C3 in MUT-KO cells (Figure 2a). The *p*-value of the difference between MUT-KO versus WT from the *t*-test was <0.001 for both the metabolites.

### 2.3. MUT Knockout Does Not Affect Cell Viability and Proliferation

In order to evaluate whether the MUT knockout could impact cell viability and growth rate in the cell cultures, we performed two types of cell viability assays: Neutral-red (NR) and MTT. The first is based on the endolysosomal functionality, while the second one on the mitochondrial functionality [11]. The NR and MTT assays showed no significant difference in viability between WT and MUT-KO cells at a 0-h time point (Figure 2b,c). Moreover, the NR assay showed no significant difference in the proliferation rate between MUT-KO and WT cells at 24-, 48-, and 72-h time points (Figure 2b), while MTT showed a slight decrease (*p* = 0.040) of absorbance in MUT-KO cell samples only at 72 h (Figure 2c).

### 2.4. Label-Free Quantification for the Proteomic Analysis of MUT-KO versus WT

**Figure 3:**
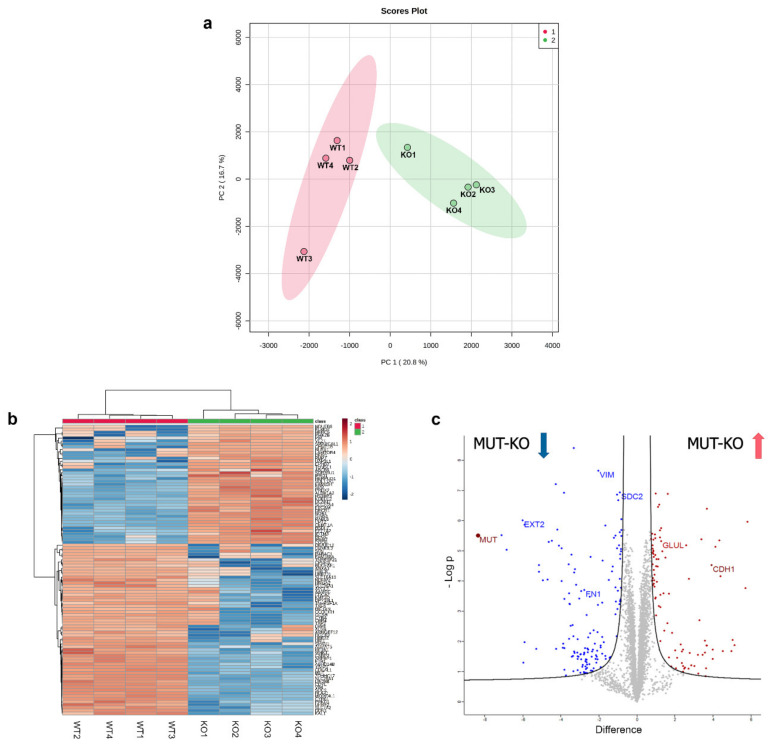
Graphic representation of the label-free proteomic experiment. Protein intensities calculated by label-free quantification (LFQ) were imported into the MetaboAnalyst 4.0 tool for the generation of the (**a**) principal component analysis (PCA) plot and (**b**) heatmap. *x*- and *y*-axis of the PCA plot report PC 1 and PC 2 that define 20.8% and 16.7% of the total variance, respectively. (**c**) The volcano plot for the visualization of the global proteome distribution in MUT-KO was obtained by plotting the log2 protein difference between MUT-KO and WT on the *x*-axis against the statistical significance, reported on the *y*-axis as -Log p. Blue and red dots represent the statistically significant down- and upregulated proteins, respectively. MUT can be visualized in the left part of the graph, indicated by a dark-red dot. The volcano plot shows by their name some significant proteins that were discussed along the manuscript (VIM: Vimentin; EXT2: Exostosin-2; SDC2: Syndecan-2; FN1: Fibronectin; GLUL: Glutamine synthetase; CDH1: E-cadherin).

In order to determine the quantitative alteration in protein expression due to MUT deficiency, we performed a proteomic experiment based on a label-free quantification (LFQ) by comparing MUT-KO versus WT cells. MUT-KO and WT groups were analyzed by the principal component analysis (PCA) showing a good separation between the two groups, as well as confirmed by the heatmap overview (Figure 3a,b). In total, the LFQ experiment led to the identification of 4341 proteins. A statistical analysis resulted in the identification of 243 differentially regulated proteins, of which 150 downregulated and 93 upregulated in the MUT-KO condition. The statistically significant proteins were graphically represented in a volcano plot (Figure 3c), highlighting in blue and red the significant down- and upregulated proteins in MUT-KO, respectively. Amongst these proteins, MUT was uniquely identified in the WT and not in the MUT-KO, confirming once again the data obtained in our cell model through WB detection (Figure 1c and Supplementary Figure S2). In addition, the volcano plot shows other significant proteins (vimentin, VIM; exostosin-2, EXT2; syndecan-2, SDC2; fibronectin, FN1; glutamine synthetase, GLUL; E-cadherin, CDH1) that will be discussed below.

### 2.5. Bioinformatic Analysis: GO Terms Annotation

**Figure 4:**
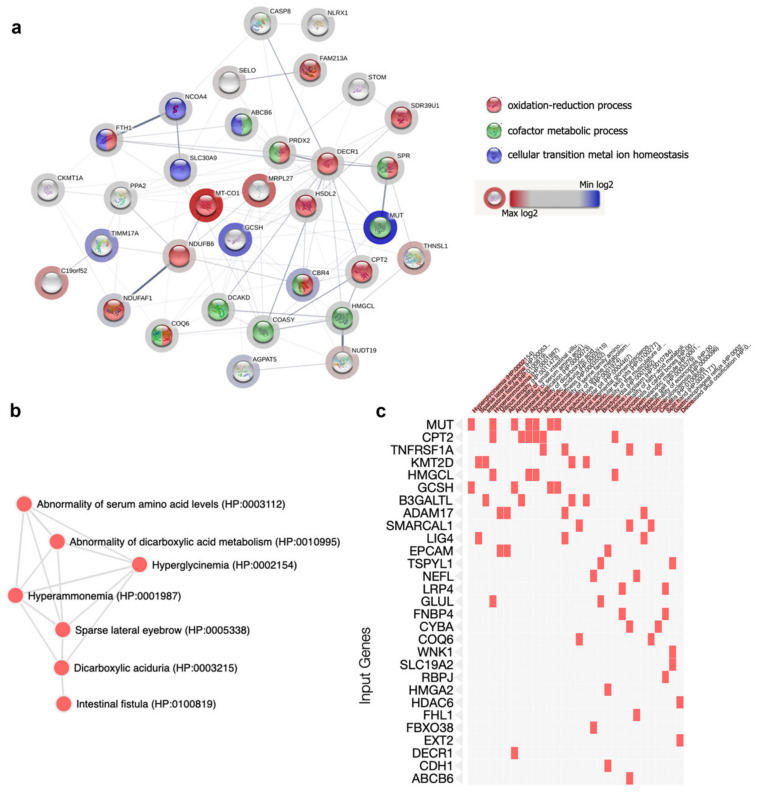
Bioinformatic enrichment of mitochondrial protein-protein interaction network and phenotypic abnormalities. (**a**) From the global list of differentially regulated proteins, those belonging to mitochondria were analyzed by STRING in order to visualize their connections and the most significant processes. Nodes colour corresponds to the enriched categories. The halo around the node has a colour ranging from blue to red, which indicates the minimum and the maximum of the log2 difference, respectively. Regulated proteins enriched (**b**) a network and (**c**) a cluster in which the most relevant phenotypic traits related to MMA emerged using the human phenotype ontology.

To get more insights into the altered molecular mechanisms in our MUT-KO model, the differential up- and downregulated proteomic datasets were analyzed using EnrichR, providing significant gene ontology (GO) terms, according to the *p*-value ranking parameter, with respect to the biological process, molecular function, and cellular component. The first 10 most statistically significant GO terms per category were selected (Supplementary Figure S3 and Table S2). Amongst these, the most interesting categories from the down- and upregulated datasets were reported in Table 1. According to the results on cellular component from the EnrichR analysis, 25 out of the 93 upregulated proteins (the 27% of the upregulated proteome) were recognized as belonging to the mitochondrial compartment after interrogation of the human MitoCarta2.0, which is an inventory that contains genes/proteins with a strong support of the mitochondrial origin or localization [12]. On this basis, also the downregulated proteomic dataset was intersected with MitoCarta2.0. Globally, 33 out of 243 proteins (the 13.6% of the differential proteome) showed mitochondrial localization or could be related to mitochondria (Table 2). Thus, we used the STRING software to analyze these 33 proteins and the resulting interaction network was reported in Figure 4a. Of note, the GO sub-analysis enriched the oxidation-reduction process (FDR 6.1 × 10^−7^), cofactor metabolic process (1.5 × 10^−5^), and cellular transition metal ion homeostasis (FDR 0.0027) as biological process categories.

In addition, the global list of differentially regulated proteins was analyzed using the human phenotype ontology (HPO), a standardized vocabulary of phenotypic abnormalities. The HPO enrichment analysis retrieved the relationship between proteins and phenotypic alterations of the disease (Figure 4b,c). Accordingly, some of the most relevant and known clinical symptoms/phenotypes related to MMA were enriched from the MUT-KO differential proteome, such as abnormality of serum amino acid levels (HP:0003112), abnormality of dicarboxylic acid metabolism (HP:0010995), hyperglycinemia (HP:0002154), and hyperammonemia (HP:0001987).

### 2.6. Rescuing MUT Protein Expression in a MUT-KO HEK 293 Cell Line

**Figure 5:**
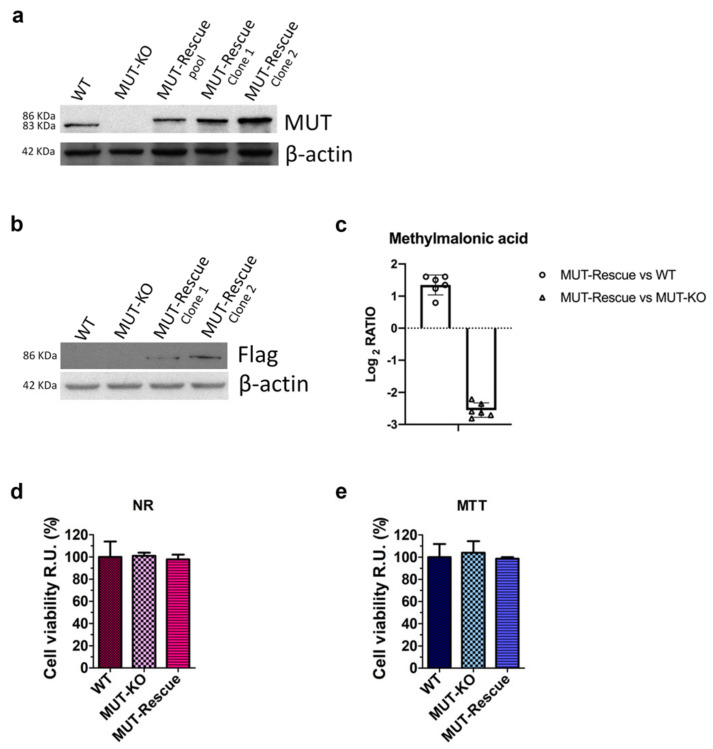
Western blot and cell viability analyses of MUT-KO cells after a stable transfection-mediated MUT expression rescuing. (**a**) WB analysis of the whole Flag-MUT plasmid-transfected MUT-KO clone 2 cell population after selection with hygromycin (MUT-Rescue pool) and two single cell clones (namely, MUT-Rescue clone 1 and 2) isolated from the MUT-Rescue pool. (**b**) MUT-Rescue clone 1 and 2 were also analyzed with an anti-Flag antibody to confirm the expression of recombinant Flag-MUT. WT and MUT-KO were used as a control; β-actin was used as a loading control. (**c**) Methylmalonic acid was measured by targeted LC-MS/MS in MUT-Rescue and compared to WT and MUT-KO levels. Concentrations were normalized to the protein content of each sample and calculated as µM based on 1 mg of protein per cell extract. Data are reported as the mean of six biological replicates ± SEM. The *p*-value for the comparisons is ≤0.001. Comparison of cell viability in WT, MUT-KO, and MUT-Rescue cells by (**d**) Neutral-red (NR) and (**e**) MTT assays. The experiments were performed in four independent biological replicates. Absorbance values were normalized to those of WT cells (used as a control) and were, hence, expressed as cell viability percentage relative units (RU). Data were reported as averages of four biological replicates and error bars represent SD. A one-way two-tail paired *t*-test was used to calculate the statistical significance of differences between the three groups. However, no statistically relevant difference was observed.

In order to establish a cellular system to be used as the control for the specificity of MUT-KO effects, we stably transfected MUT-KO cells with a plasmidic construct able to give ectopic expression of recombinant MUT protein fused at its N-terminal with a Flag epitope. In the weeks following the transfection, the pool of resistant cells (MUT-Rescue pool) was properly diluted and plated, in order to have independent colonies each formed by a single resistant cell clone. The pool and the single clones (MUT-Rescue clones 1 and 2) were analyzed by WB showing a strong expression of MUT when compared with HEK 293 WT and MUT-KO cells (Figure 5a). Clones 1 and 2 were also tested for the presence of Flag epitope fused to the MUT recombinant protein and showed the expected Flag signal (Figure 5b). Due to the higher expression, clone 2 was tested by qRT-PCR showing a significant recovery of MUT mRNA expression (Supplementary Figure S1) and chosen to be used for the following experiments. Hereinafter, clone 2 will simply be indicated as MUT-Rescue. Moreover, the levels of methylmalonic acid were also measured in MUT-Rescue samples. Targeted LC-MS/MS showed that methylmalonic acid levels in MUT-Rescue are comparable with WT cells and decreased with respect to MUT-KO, reported by the log2 ratio as 1.3 and −2.6, respectively (Figure 5c). The *p*-value for the comparison of MUT-Rescue versus WT and MUT-KO was <0.001.

In addition, in order to evaluate whether MUT rescuing could have influenced cell viability, we performed NR (Figure 5d) and MTT (Figure 5e) assays on WT, MUT-KO, and MUT-Rescue cells, respectively. The comparison of cell viability in MUT-Rescue with the other two cell types shows no significant difference (Figure 5d,e).

### 2.7. Validation of Proteomic Identifications in WT and MUT-KO and Protein Expression Levels Analysis in MUT-Rescue Cells

**Figure 6:**
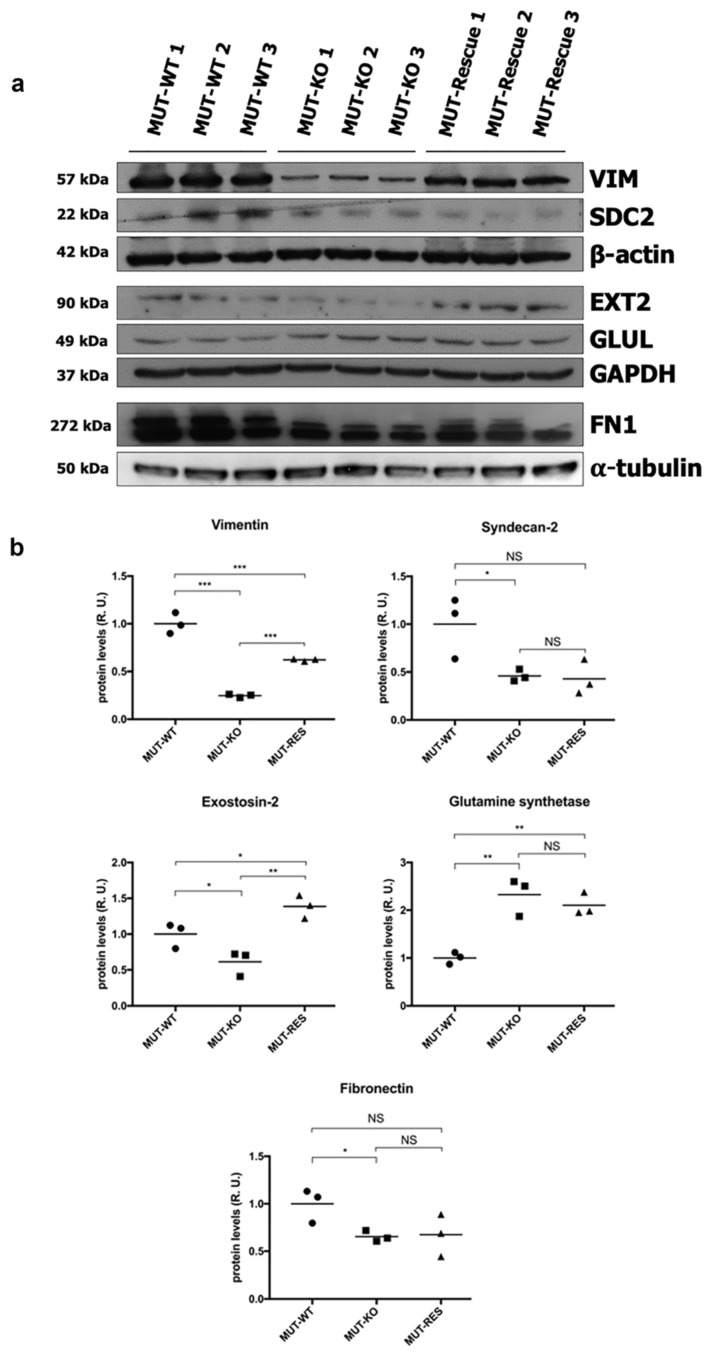
Validation of proteomic results and expression levels analysis in MUT-Rescue cells. According to the results of the proteomic LFQ, the downregulation of VIM, SDC2, EXT2, FN1, and the upregulation of GLUL were confirmed in MUT-KO cells by WB. The expression levels of these proteins were also measured in MUT-Rescue cells. (**a**) The panel reports blot scans of X-ray films for each tested protein and their loading controls. (**b**) Densitometry analysis results are reported as the mean ± SD of three biological replicates each normalized to the amount of α-tubulin, β-actin, or GAPDH proteins. A one-way two-tail *t*-test was used to calculate the statistical significance of differences between the three groups (*p* value). *: *p* < 0.05; **: *p* < 0.01; ***: *p* < 0.005; NS: Not significant (*p* > 0.05).

According to the hints derived from the bioinformatic analysis, six proteins of the differential dataset (Figure 3c) were selected because participating in processes that regulate cell structure and morphology (except for GLUL), as a novel path to investigate for the cellular damage in MMA. Thus, the identification and the quantitative abundance of these proteins were tested by WB and validated. Accordingly, the significant downregulation of VIM, SDC2, EXT2, FN1, and the upregulation of GLUL were confirmed by the densitometry analysis in MUT-KO (Figure 6a,b). The WB analysis carried out employing also the MUT-Rescue cell model revealed that protein levels were restored for VIM and EXT2 but not for SDC2, GLUL, and FN1 (Figure 6a,b). These results show that changes in the levels of VIM and EXT2 are linked to MUT deficiency in the cell model.

**Figure 7:**
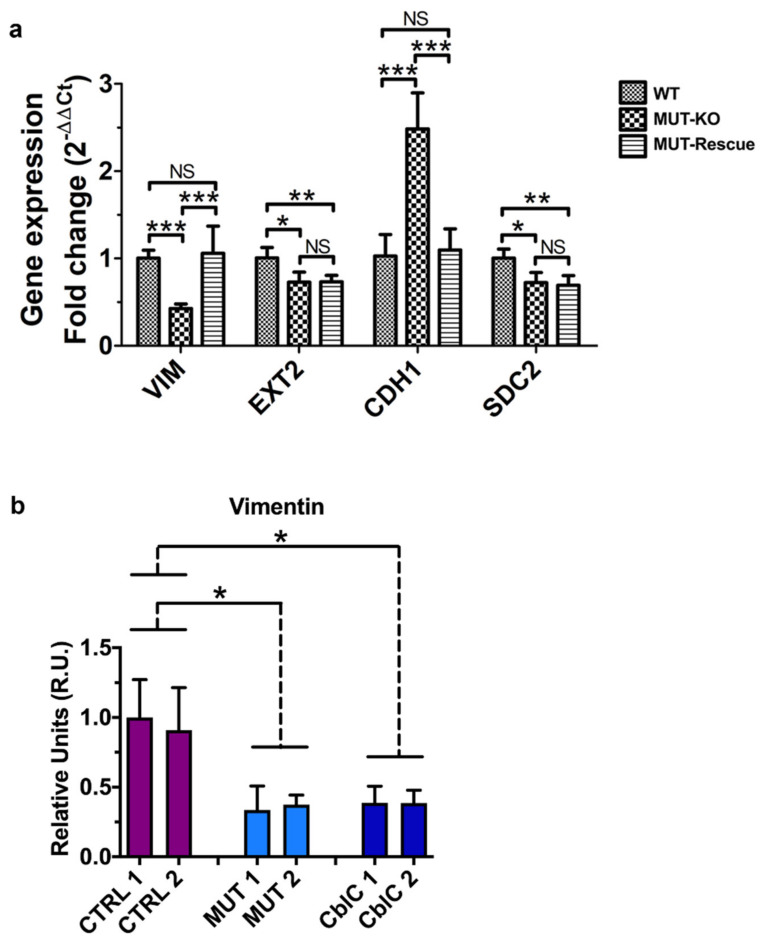
Gene expression analysis of validated proteins and immunodetection of VIM protein in the urine from patients. (**a**) Transcript levels of the proteins validated by the WB analysis were measured by qRT-PCR in the analyzed cell lines. Relative gene expression levels were normalized to those of RNA polymerase II, α-tubulin, and β-actin genes and calculated using the 2^−ΔΔCt^ method and reported as fold-change. Bars are the mean of four biological replicates while bar errors indicate SD. Statistical significance was calculated by a one-way two-tail unpaired *t*-test; *: *p* < 0.05; **: *p* < 0.01; ***: *p* < 0.005; NS: Not significant (*p* > 0.05). (**b**) Vimentin expression was tested by WB in the urine of MMA patients (MUT). Healthy donors (CTRL) were used as controls; MMA with homocystinuria CblC type patients (CblC) were used as an additional control. Bars are the mean of three independent experiments; bar errors indicate SEM. Statistical significance of MUT and CblC groups was calculated with respect to CTRL by a one-way two-tail unpaired *t*-test; *: *p* < 0.05.

In addition to protein validations, we also checked the transcript levels by qRT-PCR analysis. Only significant results were reported (Figure 7a). A diminished expression of VIM, EXT2, and SDC2 was observed in MUT-KO cells also at the gene level (Figure 7a). Coherently to the WB analysis, transcript levels of VIM are restored in MUT-Rescue; on the other side, gene expression levels are not rescued for EXT2 and SDC2.

Since we did not get clear results by WB probably due to a low quality of the antibody used, we validated CDH1 by qRT-PCR. This resulted in the increased expression of CDH1 in MUT-KO, while a normal gene expression was recovered after MUT rescuing (Figure 7a). This could imply that the observed differences in the expression of VIM and CDH1 proteins actually depend on a transcriptional regulation. On the contrary, the absence of an overlap between the trends of gene and protein expression suggests that the regulation of EXT2 or SDC2 may be controlled at the protein level.

Finally, VIM expression was tested in the urine of MMA patients (MUT). Healthy individuals (CTRL) and MMA with homocystinuria CblC type patients (CblC) were used as controls for VIM immunodetection (Figure 7b). For each individual the results obtained from three independent experiments were averaged. A diminished expression of VIM was found in the MUT group and, surprisingly, in the CblC one, both with respect to CTRL. This may indicate the involvement of VIM also in the Cbl-related MMA forms representing the result of a common alteration for both disorders.

### 2.8. MUT Knockout Increases the Intracellular Levels of ROS

**Figure 8:**
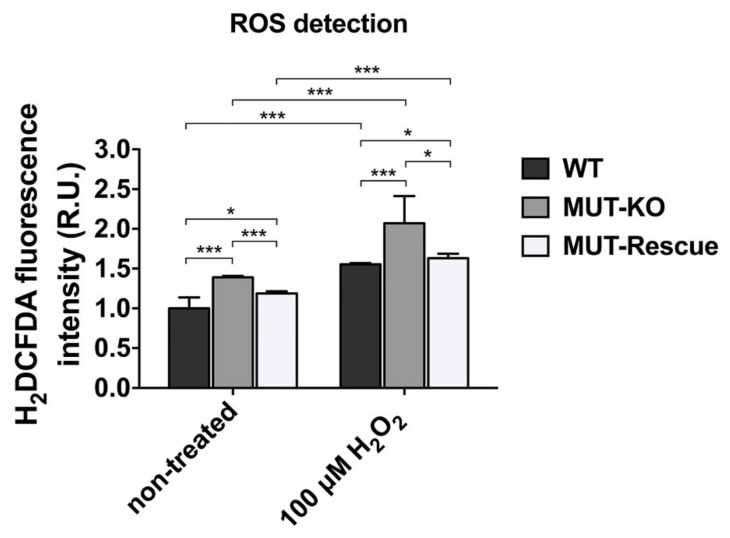
Determination of intracellular ROS levels. Non-treated and H_2_O_2_-treated WT, MUT-KO, and MUT-Rescue cells were analyzed by the H_2_DCFDA fluorescence assay. Fluorescence intensity values were obtained through three technical measures in three independent biological replicates. The averages of technical replicates measures were normalized to the total protein amount of each sample. Normalized values were expressed as fold change (relative units, RU) with respect to WT control cells and reported as mean values of the three replicate experiments whereas error bars represent SD. A one-way two-tail paired *t*-test was used to calculate the statistical significance of differences between the three cell types and in both treatment conditions. *: *p* < 0.05; ***: *p* < 0.005.

Hints from the bioinformatic analysis on the MUT-KO proteome suggested the involvement of “oxidation-reduction process” and “oxidoreductase activity”. In order to get indications about the variation of the redox state and the oxidative stress levels in our MUT-KO cell model, we measured the intracellular levels of Reactive Oxygen Species (ROS) generated by cells using the H_2_DCFDA fluorescence assay. This latter was performed in WT, MUT-KO and MUT-Rescue cells in native conditions and after incubation with H_2_O_2_ at the concentration of 100 µM in order to induce additional oxidative stress (Figure 8). Data clearly reveal that ROS detection is significantly higher in MUT-KO cells in native conditions if compared to WT and MUT-Rescue ones. The intracellular levels of ROS resulted increased after incubation with H_2_O_2_, thus suggesting that the MUT-KO cells are more sensitive to oxidative stress.

### 2.9. MUT Knockout Impairs Cell Viability and Mitochondrial Functionality in a Propionate-Enriched Culture Medium

**Figure 9:**
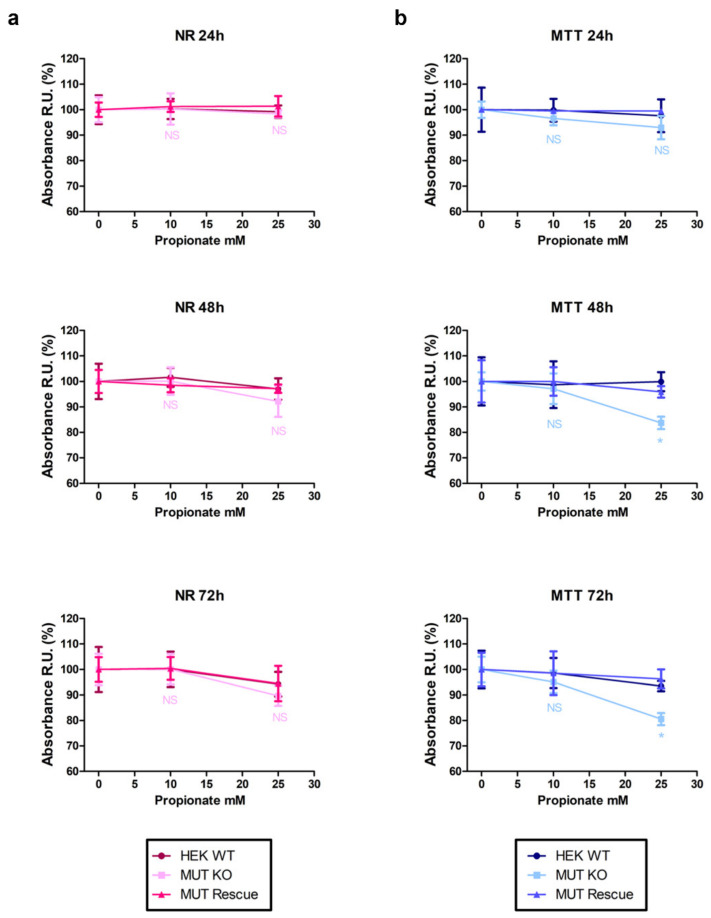
Cell viability assays in a propionate-enriched culture medium. WT, MUT-KO, and MUT-Rescue cells were kept in culture in a propionate-enriched medium and analyzed by (**a**) NR and (**b**) MTT assays. The analyses were performed in three independent biological replicates. Absorbance values for each cell type were expressed as fold change (relative units, RU) with respect to the values of the corresponding non-treated (0 h time point) cell type. Data were reported as mean values of the three experimental replicates ± SD. A one-way two-tail paired *t*-test was used to calculate the statistical significance of differences between the three tested cell types. * *p* < 0.05; NS: Not significant (*p* > 0.05).

Previous studies on cells carrying defects in the *MUT* gene have shown that the addition in the culture medium of metabolic precursors of methylmalonyl-CoA (e.g., propionate) highlights pathway unbalances [11]. Here, we used a propionate-enriched culture medium to investigate whether, under these conditions, MUT knockout could influence cell viability and mitochondria functionality. To this aim, we compared the results of paralleled NR (Figure 9a) and MTT (Figure 9b) assays. Actually, no significant difference in the NR uptake levels was observed between propionate-treated cells and the respective untreated controls (e.g., propionate-incubated MUT-KO vs. untreated MUT-KO—0 h time point). Moreover, results from this assay showed no significant differences between the three cell types (WT, MUT-KO, and MUT-Rescue) at each tested propionate concentration (10 and 25 mM) and time point (24, 48, and 72 h). Accordingly, a 10 mM concentration showed no significant effect even in the MTT assay, while 25 mM of propionate at 48 and 72 h caused a significant reduction in MTT absorbance of MUT-KO cells when compared with untreated controls (0 h time point) and with the other cell types (WT and MUT-Rescue). These results imply that 25 mM of propionate is able to induce reduction of mitochondrial functionality (succinate dehydrogenase activity) in MUT-KO cells while this effect is clearly restored in MUT-expressing cells.

### 2.10. MUT Knockout Hampers Cell Structure and Morphology Impacting on Proteoglycans and Glycoproteins

**Figure 10:**
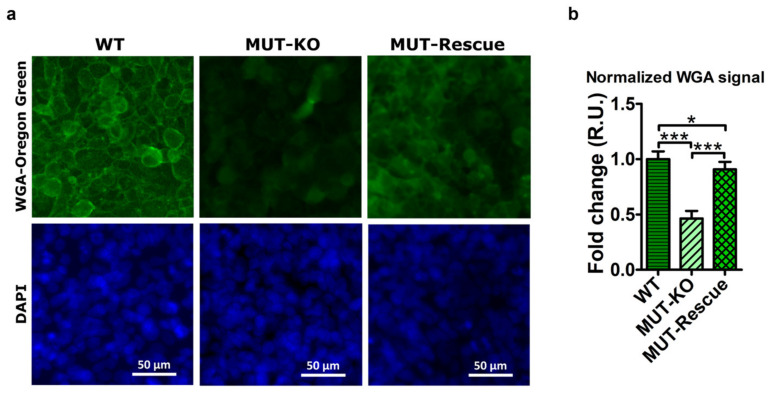
Microscopy analysis of fluorescent wheat germ agglutinin (WGA) staining in HEK 293 and genome-modified cells. (**a**) Microscopy images of WT, MUT-KO, and MUT-Rescue cells after staining with Oregon Green-labeled WGA and with DAPI. Cells were observed with a Leica DMI 4000 B inverted microscope equipped with Leica GFP and DAPI filter cubes, by using a 40× objective and images were acquired with the Leica LAS AF software. (**b**) Quantitative analysis of fluorescent signals was performed in three independent biological replicates and three microscope fields were acquired for each biological replicate. The signal intensity of acquired micrographs was measured by the ImageJ software. WGA signals were normalized to DAPI signals and expressed as fold change (relative units, RU) with respect to values of WT cells. Data were reported as the mean values of three biological replicates and error bars represent SD. A one-way two-tail paired *t*-test was used to calculate the statistical significance of differences between the three tested cell types. *: *p* < 0.05; ***: *p* < 0.005.

Proteomic analysis in MUT-KO revealed reduced levels of EXT2 (validated both at the protein and gene level), which is one of the two glycosyltransferases involved in the chain elongation step of heparan sulphate biosynthesis, and downregulation of two additional glycoproteins, SDC2 and FN1. Actually, MUT reintroduction produced an increase of EXT2 only at the protein level (Figure 6a,b and Figure 7a). In order to further investigate on the role of glycoproteins, a microscopy analysis was performed in WT, MUT-KO, and MUT-Rescue cells, after staining with fluorescently-labeled wheat germ agglutinin (WGA). Being this lectin known to bind to sialic acid and N-acetylglucosaminyl residues, it is, as an example, adopted to mark the glycocalyx of endothelial cells [13]. Hence, MUT-KO cells show a significantly lower fluorescent-WGA signal when compared with WT, while the rescuing of MUT expression seems to partially revert this effect, even though in MUT-Rescue such a signal remains slightly lower than in WT cells (Figure 10b). Moreover, also the staining pattern resulted as different in the three cell types (Figure 10a). In WT cells, the appearance of WGA staining is very definite and it delimits sharply the cell borders. On the contrary, the staining appears much more diffuse in MUT-KO cells, while in MUT-Rescue it seems halfway between the other two cell types. These phenomena suggest that the MUT knockout could impair the morphology and structure of the cell impacting on either the distribution and organization of glycoproteins or the synthesis of their glyosidic chains, or both.

## 3. Discussion

To investigate the mechanisms of damage which occurs in methylmalonic acidemia, we developed a MUT-KO HEK 293 cell line. Being aware that a cultured cell system cannot fully predict the in vivo responses of complex living systems, HEK 293 were chosen for being highly transfectable cells, monitoring over the time the stability of the knockout. Although the cells were viable, methylmalonic acid and propionylcarnitine, diagnostic markers for MMA, were increased. These abnormalities were reversed by transfection of wild type *MUT* cDNA. Proteomic and bioinformatic analyses were employed to address protein changes to altered pathways in the MUT-KO cell model, as summarized below.

### 3.1. MUT-KO and Cell Architecture Modification: Cytoskeleton, Cell Adhesion, and Cell Junction Organization

Differential abundance in cytoskeletal proteins is evinced in the MUT-KO proteome, suggesting that processes related to extracellular matrix deposition, cytoskeleton organization, cell junctions, and cell communication were altered as a consequence of MUT knockout. In order to investigate such alterations, WGA staining revealed an important loss of fluorescence in MUT-KO compared to WT, with a confused and diffused cell staining pattern that is suggestive of structural or morphological modifications that impact cell-cell junctions and cell architecture, probably impairing glycoproteins distribution and organization. Interestingly, the main proteins related to cell architecture modification were included in Table 3. Among these, EXT2 is striking because of its strong quantitative decrease in MUT-KO, also confirmed at the gene level. However, EXT2 protein levels are completely recovered in MUT rescuing experiments, even though mRNA levels do not change. Thus, we noticed that the WGA fluorescence intensity was recovered after MUT rescuing, while its staining pattern did not delimit very sharply the cell borders not resulting as definite as well as WT cells. This can explain that the structural alteration due to MUT deficiency is sorely impaired and cannot be totally recovered by MUT itself or by the rescue of other dysregulated proteins.

As for EXT2, a similar behaviour was exhibited by VIM, whose downregulation results were restored in MUT-Rescue at both the gene and protein level. In addition, VIM expression was found low in the urine of one MMA patient and in two MMA with homocystinuria CblC type patients. This finding may indicate the involvement of VIM as a target protein common to both isolated and CblC-related MMA forms. Since the rarity of MMA, the poor availability of patient specimens did not allow us to extend this analysis to a wider cohort for a deeper investigation. In line with this, Hannibal et al. [14] identified an important downregulation of VIM in a proteomic experiment on fibroblasts from CblC patients. Being involved in cytoskeleton formation and regulation of integrin functions, we can speculate that VIM downregulation contributes to inducing cytoskeleton reorganization by modulating cell adhesion and cell-cell connections, with an impairment of normal cell structure and functions [15,16]. Accordingly, the downregulation of cytoskeletal and junction proteins has emerged in vimentin-silenced cell lines [17]. Thus, VIM and EXT2 may have an important role in the structural alterations present in MUT-KO cells. In accordance with these results, we found reduced levels of CGN, which plays a role in the formation of tight junctions, and NCAM1 and CYR61, which could contribute in the impairment of the structural organization of the cell by defective adhesion mechanisms.

VIM, FN1, and CDH1, which were found dysregulated in MUT-KO, represent markers of mesenchymal-epithelial transition (MET), involved in many developmental and disease processes. Zeisberg et al. [18] discovered that renal fibroblasts can be induced into MET to form epithelial aggregates able to repair kidney tubular injury. Since MET is a key process in nephrogenesis and is associated with reprogramming for the establishment of epithelial polarity, metabolic switching, and epigenetic modifications even in somatic cells [19], the perturbation in these three proteins in MUT-KO cells may represent an additional link between MMA and renal injury. In addition, it is interesting to observe that MUT-KO cells showed also downregulation of ANXA3. It has been reported [20] that MET is induced by knocking down ANXA3 by increasing epithelial markers (CDH1, γ-catenin) and decreasing mesenchymal markers (VIM, N-cadherin).

Cytoskeletal alterations are also confirmed by the downregulation of EPS8, an actin-binding protein that regulates both dynamics and the organization of actin cytoskeleton. Specifically, it was demonstrated that Eps8 enhances neuronal cell proliferation and migration through the PI3K-Akt pathway and increases β-catenin levels with modulation of cell-cell adhesion, mimicking the Wnt pathway [21,22,23]. In accordance with this speculation, we found downregulation of β-catenin in a previous cell model of MUT-knockdown [11]. The above-described alterations in the MUT-KO proteome reflect the consequence of the MUT absence affecting cell adhesion and cell connections as a direct cause of damage and decompensation. Moreover, since cells did not show a total recovery in the Rescue experiment and cell viability is not perturbed, MUT-deficient cells may modulate cell architecture as an adaptative mechanism.

### 3.2. Metal Homeostasis Unbalances in MUT-KO

In addition to the structural modifications, MUT-KO cells showed a set of dysregulated proteins involved in metal homeostasis (Table 4). In fact, cell component categories enriched in the MUT-deficient proteome, “autophagosome“ and “autolysosome”, included a set of key proteins characterized by a central role in autophagic processes, such as ferritinophagy. In this context, the quantitative changes of FTH1 and NCOA4 depend on iron levels, even the perfect correlation is still unknown [24]. When iron levels in the cell are low, ferritinophagy (the autophagic degradation of FTH1) is selectively mediated by NCOA4, whose expression is increased in order to target FTH1 to the lysosome for iron release. In contrast with this, when ferritinophagy occurs both FTH1 and NCOA4 are degraded into the lysosome, reducing the levels of both proteins [24]. The degradation of iron-containing macromolecules such as FTH1 into lysosomes increases the release of iron that can catalyze the production of ROS via Fenton reaction [25,26]. Ferritinophagy could even result in a reduced iron supply for enzymes (such as cytochromes) which can impair the oxidative phosphorylation. With these hypotheses, the downregulation of FTH1 and NCOA4 in the MUT-KO proteome highlights unbalances in iron homeostasis that may contribute to ROS production, providing evidence for an additional mechanism of cellular damage related to MUT absence. Particularly, in the study of brain damage, NCOA4-mediated ferritinophagy is supposed to link iron homeostasis unbalances and autophagy to neurodegeneration [27]; accordingly, this mechanism may be linked to neuronal damage in MMA patients. Moreover, the downregulation of FTH1 may be directly related to an increase in the level of IREB2 protein, which was demonstrated to repress FTH1 expression by binding to iron-responsive elements in ferritin mRNA [28]. In addition, the MUT-KO proteome showed increased levels of ABCB6, a mitochondrial transporter involved in porphyrin transport, iron homeostasis, or resistance to cytotoxic agents [29,30], which may be induced by increased levels of iron into the cells. Finally, the bioinformatic analysis showed an enrichment in the class of proteins characterized by “divalent inorganic cation transmembrane transporter activity” including SLC39A6, SLC39A10, SLC39A1, and ZDHHC17. These findings reveal that the metal homeostasis is affected in MUT-deficient cells and represents one other possible cause of damage underlying MMA.

### 3.3. Mechanisms of Stress Linked to MUT: ROS, Hyperammonemia, and Propionate

In order to investigate on the MUT involvement in pathways whose perturbation is directly responsible for the stress in the cell, we proved that ROS were significantly increased in MUT-KO whereas the rescue of MUT protein was able to restore normal levels. Furthermore, we found that a relevant increase in ROS levels occurs in MUT-KO cells even in the case of additional stress induced by H_2_O_2_ and, again, ROS levels were comparable to WT cells after MUT reintroduction. This proves a direct correlation between MUT absence and the elevation of ROS levels in MUT-KO cells, suggesting a higher susceptibility to stressing events that causes ROS overproduction. This behaviour is strongly in line with the knowledge that ROS and oxidative processes are unbalanced in MUT-deficient patients and responsible for most of the damage of MMA [31,32,33].

Moreover, hyperammonemia is a major complication of MMA, especially in the brain and liver [34]. To prevent hyperammonemia, glutamate and ammonia react to form glutamine, in a reaction catalyzed by GLUL [35]. Here, an increase in the levels of GLUL protein was validated in MUT-KO, thus providing a role for this enzyme in protecting cells from ammonia toxicity provoked by MUT KO. Actually, our proteomic investigation did not allow us to find enzymes involved in ammonia production. However, the increased GLUL expression was not rescued at normal levels after MUT reintroduction, thus convincing that this aspect, corroborated by recent findings revealing that the treatment with glutamine protected from ROS and oxidative damage [36], needs further investigation. Thus, GLUL upregulation could be induced for protecting cells from ammonia accumulation via incorporation in glutamine and, at the same time, providing protection against oxidative damage.

Even if cell viability is not perturbed by the MUT knockout, mitochondrial functionality was shown by the MTT assay to be compromised after stressing cells with propionate, a precursor metabolite of methylmalonyl-CoA, suggesting that the MUT knockout increases the sensitivity of the cells to this stress. Since the rescue of the protein permits the cell to resist to propionate-induced stress, we directly address the susceptibility to the cellular damage to the absence of MUT. This increased susceptibility to stress is reasonable with the fact that metabolic instability in patients has the tendency to worsen, sometimes even after organ transplantation [37,38].

### 3.4. Mitochondrial Alterations in MUT-KO

Despite the use of stressing molecules precursors of MUT activity, MTT experiments in MUT-KO showed a slightly decreased mitochondrial succinate dehydrogenase activity even in the absence of propionate, implying basal mitochondrial alterations. In fact, structural/morphological (i.e., enlarged mitochondria called megamitochondria, or fragmented cristae) and functional (i.e., alteration of mitochondrial membrane potential and energy metabolism) abnormalities are triggered downstream of MUT deficiency by accumulating toxic metabolites within the mitochondrial matrix [33]. Accordingly, the proteomic analysis revealed that several mitochondrial proteins result as quantitatively altered (Table 2), reinforcing the findings of mitochondrial alterations in MUT-KO cells. Then, the bioinformatic analysis of dysregulated mitochondrial proteins showed altered pathways related to energy deficiency and metals and redox homeostasis in the MUT-KO cell model, features only in part known for MMA patients. In addition to the abovementioned FTH1, NCOA4, ABCB6, and SLC30A9 (Table 4), the main proteins related to mitochondrial alterations were included in Table 5. The increased level of HMGCL that produces acetyl-CoA and acetoacetate can be linked to the enhancement of ketogenesis, a feature of MMA patients [39,40]. Indeed, we also found increased levels of COASY, which is involved in CoA biosynthesis [41]. CPT2 is a nuclear protein transported to the mitochondrial inner membrane where, together with CPT1, transports long-chain fatty acids in the mitochondrion for subsequent oxidation. While defects of CPT2 are often causative of fatty acid oxidation disorders [42], its upregulation may probably reflect the demand of delivering long-chain fatty acids to the mitochondrial matrix as fuel for the cell; in concordance, the upregulation of SLC27A4 and DECR1 may suggest an increased rate in fatty acid oxidation. Furthermore, the upregulation of CPT2 was found to increase ROS, contributing to cellular damage [43]. The upregulation of NUDT19 may be linked to an increase in the hydrolysis of fatty acyl-CoA esters. Finally, the downregulation of GCSH is in line with the study of Kølvraa [44], explaining the hyperglycinemia found in methylmalonic and propionic acidemias as induced by branched-chain amino acids through the inhibition of the glycine cleavage system. These results confirmed our previous speculations on altered mitochondrial functionality on a MUT-knockdown cell model [11] and was clearly established by recent literature [33].

### 3.5. Intracellular Trafficking in MUT-KO

Proteomic and bioinformatic analysis suggested alteration of vesicular trafficking in MUT-KO cells. Actually, these data revealed downregulation of proteins belonging to the phagocytic vesicle membrane, Golgi subcompartment, autophagosome, autolysosome (Table 1 and Supplementary Table S2) and can be linked with the observed mitochondrial unbalances. In fact, there is mounting evidence that degenerated mitochondria are targeted by selective autophagy (mitophagy), mediated by specific ubiquitin ligase activities, and that these mechanisms may escape in MMA [33]. Interestingly, among autophagosome-related downregulated proteins we found ubiquitin carboxyl-terminal hydrolase 33 (USP33), a deubiquitinating enzyme which is reported to be involved in various processes including the regulation of autophagy [45]. Accordingly, in the same category appears the serine/threonine-protein kinase D1 (PRKD1), a protein kinase with several roles in angiogenesis, apoptotic process, inflammatory response, innate immune response, and intracellular signal transduction [46]. PRKD1 also acts in the positive regulation of autophagy in response to oxidative stress. Particularly, in conditions of oxidative damage the activity of PRKD1 is enhanced in order to activate autophagy by inducing autophagosome formation [46]. The downregulation of these proteins in MUT-KO proteome suggests how an important mechanism such as autophagy may not be functioning in MMA, reinforcing the idea of autophagy (mitophagy) dysfunction in metabolic disorders of branched-chain amino acid and fatty acid metabolism [47]. Thus, the overall proteomic investigation is in line with the recently reported findings about compromised mitophagy of degenerated mitochondria in MUT deficiency [33].

## 4. Material and Methods

### 4.1. Cell Cultures and Treatments

HEK 293 cells (human embryonic kidney, ATCC no. CRL-1573) were cultured with high glucose in Dulbecco’s Modified Eagle Medium (DMEM) (EuroClone, Paington, UK) supplemented with a 15% fetal bovine serum (EuroClone, Paington, UK), 4 mM L-Glutamine (Sigma-Aldrich, St. Louis, MO, USA), 1% of the penicillin-streptomycin solution (Sigma-Aldrich, St. Louis, MO, USA) at 37 °C in a 5% CO_2_ atmosphere.

For the propionate treatment, 0.5 × 10^3^ cells/mm^2^ were seeded into the wells of a 24-well microplate (Costar, Corning Inc., Corning, NY, USA). After 24, 48, and 72 h, the medium was replaced with a fresh medium containing 10, 25, 50 mM of sodium propionate (Sigma-Aldrich, St. Louis, MO, USA) and cells were analyzed in the time-course by MTT and Neutral Red assays (see below) after 24, 48, and 72 h. The values were normalized versus untreated cells at respective time points.

### 4.2. Genome Editing and Transfections

For *MUT* gene knockout by the CRISPR/Cas9 technique, 1.5 × 10^3^ cells/mm^2^ of HEK 293 wild type cells were seeded in a 10 cm diameter plate and kept in culture in a medium without antibiotics. After 24 h, cells were transfected with 12 µg of “MUT CRISPR/Cas9 KO Plasmid (h2)” (Santa Cruz Biotechnology, Dallas, TX, USA) and 12 µg of “MUT HDR Plasmid (h2)” (Santa Cruz Biotechnology, Dallas, TX, USA), using Lipofectamine 2000 (Thermo Fisher Scientific, Waltham, MA, USA) following the supplier instructions. The HDR plasmid constitutes a template for homology-directed repair (HDR) after the CRISPR-mediated double-strand break. The HDR permits the insertion in the breakpoint of a cassette which confers puromycin resistance and RFP expression. After 48 h from the transfection, the culture medium was replaced with a selective medium containing 1 µg/mL puromycin (Santa Cruz Biotechnology, Dallas, TX, USA). The transfected cell pool (MUT-KO pool) was kept in culture in a selective medium for four days, with phosphate buffered saline (PBS) (EuroClone, Paington, UK) washes and medium changes, in order to eliminate detached cells and select adherent puromycin-resistant cells. Then, MUT-KO pool cells were detached from the plate, properly diluted and seeded again in a 10 cm diameter plate with a selective medium, in order to have a few isolated cells able to form distinct colonies each constituted by a single cell clone. The colonies were then detached and kept in culture separately. MUT-KO pool and the clones (MUT-KO clone 1 and clone 2) were tested by WB to verify the absence of MUT protein expression. Expression levels of MUT mRNA were assayed in MUT-KO clone 2 by qRT-PCR analysis (Supplementary Figure S1).

In order to rescue the expression of MUT protein in MUT-knocked out cells, MUT-KO clone 2 was transfected as described above (for HEK 293 cells), with 24 µg of “pCMV3-Flag-MUT” plasmid (Sino Biological, Beijing, China). After 48 h from the transfection, the culture medium was replaced with a selective medium containing 1 µg/mL puromycin and 150 µg/mL hygromycin. The transfected cells (MUT-Rescue) were kept in culture in a selective medium and a pool of stably transfected (hygromycin-resistant) cells (MUT-Rescue pool) and distinct single (stably transfected) cell clones were obtained as described above (for MUT-KO). MUT-Rescue pool and the clones (MUT-Rescue clone 1 and clone 2) were tested by WB to verify the presence of recombinant MUT protein expression. Expression levels of MUT mRNA were assayed in MUT-Rescue clone 2 by the qRT-PCR analysis (Supplementary Figure S1).

### 4.3. Methylmalonic Acid and Propionylcarnitine Measurement

Methylmalonic acid and propionylcarnitine (C3) were measured by LC-MS/MS, as elsewhere reported [6,48]. Briefly, cells were homogenized in 500 μL of cold methanol. The supernatant was separated from proteins and cell debris by centrifugation at 14,000× *g* for 20 min at 4 °C. The supernatant was used for the detection of methylmalonic acid and C3. The analyses were performed using an API 4000 triple quadrupole mass spectrometer (Applied Biosystems-Sciex, Toronto, ON, Canada) coupled with a 1100 series Agilent high-performance liquid chromatograph (Agilent Technologies, Waldbronn, Germany). Metabolites concentrations were normalized to the protein content, and calculated based on 1 mg protein for each cellular extract and expressed as µM [49]. Data, reported as the mean of six replicates ± standard error of the mean (SEM), were elaborated using GraphPad Prism version 7.0a. Differences between treatments were considered significant at a value lower than 0.05.

### 4.4. Microscopy Analysis

For the detection of red fluorescence (excitation maximum at 558 nm; emission maximum at 583 nm) due to the red fluorescent protein (RFP) expression, the pool and the clones of MUT-KO cells were seeded in the wells of a six-well plate at a confluency of 1.5 × 10^3^ cells/mm^2^. After 24 h, the culture medium, after two washes with PBS, was replaced with Hank’s balanced saline solution (HBSS) (EuroClone, Paington, UK) without phenol red. The cells were then observed with a Leica DMI 4000 B inverted microscope (Leica Biosystems, Wetzlar, Germany), equipped with a Leica N3 filter cube (bandpass: 546 ± 12 nm excitation; 600 ± 40 nm suppression), using a 20× objective and images were acquired with the Leica LAS AF software (Leica Biosystems, Wetzlar, Germany).

For the analysis of plasmatic membrane proteoglycans, the cells were stained with Oregon Green 488-labeled (excitation maximum at 496 nm; emission maximum at 524 nm) wheat germ agglutinin (WGA) (Thermo Fisher Scientific, Waltham, MA, USA). In detail, 1.5 × 10^3^ cells/mm^2^ were seeded in a 24-well plate. After 24 h, the cells were washed twice with PBS and fixed by incubation in PBS containing 4% (*w*/*v*) paraformaldehyde (Sigma-Aldrich, St. Louis, MO, USA) for 5 min. The cells were then washed twice with PBS and incubated in PBS containing 5.0 μg/mL WGA for 10 min. After two additional washes in PBS, cells were incubated with a solution of 300 nM DAPI (Thermo Fisher Scientific, Waltham, MA, USA) in PBS for 5 min. The cells were again washed twice with PBS, covered with a solution containing Prolong Gold antifade reagent 50% (*v*/*v*) in PBS and observed with a Leica DMI 4000 B inverted microscope, equipped with Leica GFP (bandpass: 470 ± 40 nm excitation; 525 ± 50 nm suppression), and DAPI (bandpass: 420 ± 30 nm excitation; 465 ± 20 nm suppression) filter cubes, using a 20× objective. Experiments were performed in three independent biological replicates and four microscope fields were acquired from each replicate using the Leica LAS AF software. A quantitative analysis of fluorescent signals was performed with ImageJ (Image J, NIH, Bethesda, MD, USA) software and the values of Oregon Green 488 were normalized with those of DAPI.

### 4.5. ROS Assay

ROS levels were measured in HEK 293 WT, MUT-KO, and MUT-Rescue cells. In particular, 2.0 × 10^3^ cells/mm^2^ were seeded in 24-well plates. After 24 h, cells were washed twice with HBSS and incubated for 1 h (at 37 °C in a 5% CO_2_ atmosphere) in HBSS containing 10 µM 2′,7′-dichlorofluorescin diacetate (H_2_DCFDA) (Sigma-Aldrich, St. Louis, MO, USA). After 1 h, the H_2_DCFDA solution was removed and cells were washed twice with HBSS. A part of the samples was incubated for one additional hour with 100 µM H_2_O_2_, in order to induce over-production of ROS in the cells. Native and H_2_O_2_-treated cells were collected and lysed in a RIPA buffer (Sigma-Aldrich, St. Louis, MO, USA) containing protease inhibitor cocktail (Thermo Fisher Scientific, Waltham, MA, USA). Cell extracts were centrifuged at 15,000× *g* for 10 min at 4 °C. Supernatants were collected and fluorescence was read in a 96-well black plate using a Perkin Elmer Enspire plate reader (Perkin Elmer, Waltham, MA, USA) at 485 and 527 nm as excitation and emission wavelengths, respectively. The protein concentration was measured for each sample by the Bradford assay and used to normalize fluorescence levels.

### 4.6. MTT and Neutral-Red Assays

Both MTT and neutral-red (NR) assays were performed as reported elsewhere [11,50]. Briefly, the culture medium was removed from the plate and replaced with a fresh medium containing 0.5 mg/mL of MTT (3-(4,5-dimethylthiazol-2-yl)-2,5-diphenyltetrazolium bromide) or 0.33 mg/mL of the NR solution (both Sigma-Aldrich, St. Louis, MO, USA). Cells were incubated with the reagents for 2 h at 37 °C and washed with PBS in order to completely remove them. Then, for the MTT assay, a solution of 1 N hydrogen chloride-isopropanol (1:24, *v*:*v*) was pipetted to each well and mixed to dissolve the dark-blue formazan crystals formed. After a few minutes of gentle agitation on a rocking platform at room temperature, the absorbance of each sample was read at 570 nm in a Perkin Elmer Enspire microplate reader. For the NR assay, a solution of acetic acid-water-ethanol (1:49:49, *v*:*v*:*v*) was pipetted to each well to solubilize the dye and, after a few minutes of gentle agitation, the absorbance of each sample was read at 540 nm in the plate reader.

### 4.7. Western Blot

All protein samples analyzed by Western blot (WB) in this paper underwent the same procedure. Protein lysates from cells were obtained upon lysis in a RIPA buffer (Sigma-Aldrich, St. Louis, MO, USA) and centrifugation at 14,000× *g* for 30 min at 4 °C to collect the protein supernatant. For urine proteins, urine samples were collected as described [51] and concentrated using centrifugal filters Amicon Ultra-15 3k (Millipore, Burlington, MA, USA). Protein extracts were fractionated by a 10% SDS-PAGE, and transferred onto nitrocellulose membranes using a Trans-Blot Turbo Transfer System (Bio-Rad, Hercules, CA, USA). Membranes were blocked for 3 h at room temperature with 5% milk in PBS. Each primary antibody used for WB against MUT (sc-136541, Santa Cruz Biotechnology, Dallas, TX, USA), Flag (F1804, Sigma-Aldrich, St. Louis, MO, USA), Vimentin (sc-373717, Santa Cruz Biotechnology), Exostosin-2 (sc-514092, Santa Cruz Biotechnology, Dallas, TX, USA), Syndecan-2 (sc-365624, Santa Cruz Biotechnology, Dallas, TX, USA), Glutamine synthetase (sc-74430, Santa Cruz Biotechnology, Dallas, TX, USA), and Fibronectin (sc-271098, Santa Cruz Biotechnology, Dallas, TX, USA) was incubated O/N at 4 °C in 1% milk in PBS with a 0.05% Tween-20. Primary antibodies used for detection of GAPDH (sc-32233, Santa Cruz Biotechnology, Dallas, TX, USA), β-actin (ab8226, Abcam, Cambridge, UK), and α-tubulin (T6074, Sigma-Aldrich, St. Louis, MO, USA) normalizing proteins were incubated for 2 h at RT. Immunoblot detections were carried out using horseradish peroxidase-conjugated antibodies (GE Healthcare, Piscataway, NJ, USA) and enhanced chemiluminescence (GE Healthcare, Piscataway, NJ, USA). Signals were visualized after an X-ray film exposure. Images were acquired using the GS-800 calibrated densitometer scan (Bio-Rad, Hercules, CA, USA) and elaborated using the Fiji (Image J, NIH, Bethesda, MD, USA) image processing program. VIM expression was tested in the urine of two MMA patients, two MMA with homocystinuria CblC type patients, and two healthy individuals. Urine protein signals were normalized to the intensity of the respective gel lane detected over the nitrocellulose membranes by Ponceau S staining. The VIM immunoblot was technically repeated three times and the results for each patient were averaged. Statistical significance was calculated by a paired *t*-test. The WB analysis on urine from patients was performed in compliance with ethical standards and the procedures were approved by the Italian Ministry of Health in the law 167 of 19 August, 2016.

### 4.8. Quantitative Real-Time PCR

For each qRT-PCR assay, 1.5 × 10^3^ cells/mm^2^ of HEK 293 cells were seeded in a 6 cm diameter plate and kept in culture in standard conditions (see above). After 24 h, cells were washed twice with PBS, detached from the plate by trypsinization, resuspended in PBS containing 5% FBS, and centrifuged at 250× *g* for 10 min. The supernatant was discarded and total RNA was extracted from cell pellets by using a RNeasy Mini Kit (Qiagen, Hilden, Germany); 500 ng of RNA were reverse-transcribed using SuperScript™ VILO™ MasterMix (Thermo Fisher Scientific, Bremen, Germany). Then, qRT-PCR was carried out in a 7500 Real-Time PCR System PCR Thermal Cycler with appropriate primers using a SYBR^®^ Select Master Mix (Applied Biosystems, Monza, Italy). Relative gene expression levels of VIM, EXT2, SDC2, CDH1, and GLUL were normalized to RNA polymerase II (POLR2A), α-tubulin (TUBA1A), and β-actin (ACTB) and calculated using the 2^−ΔΔCt^ method, as reported elsewhere [52,53]. Average values from at least three independent experiments were graphically reported as relative units. Statistical significance was calculated by a two-tail unpaired *t*-test. Supplementary Table S1 reports the sequences of the primers used for the qRT-PCR experiments.

### 4.9. Proteomics Sample Preparation

Four biological cell samples per experimental condition were lysed for the proteomic experiment and an aliquot from each sample was taken and tested by WB in order to ensure once again the absence of MUT protein in MUT-KO samples prior to the proteomic analysis. Supplementary Figure S2 shows the total absence of MUT protein in all four MUT-KO replicates. The S-Trap^TM^ micro spin column (Protifi, Huntington, NY, USA) digestion was performed on 50 µg of proteins from a cell lysate according to the manufacturer’s protocol. Briefly, a final concentration of 5% SDS was added to the samples. Proteins were reduced with the addition of TCEP (Sigma-Aldrich, St. Louis, MO, USA) to a final concentration of 100 mM and alkylated with the addition of iodoacetamide (Sigma-Aldrich, St. Louis, MO, USA) to a final concentration of 50 mM. Aqueous phosphoric acid was added to a final concentration of 1.2%. Colloidal protein particulate was formed with the addition of six times the sample volume of a S-Trap binding buffer (90% aqueous methanol, 100 mM TEAB, pH 7.1). The mixtures were put on the S-Trap micro columns and centrifuged at 4000× *g* for 30 s. The columns were washed three times with 150 µL S-Trap binding buffer and centrifuged at 4000× *g* for 30 s between washes. Samples were digested with 2 µg of trypsin (Promega, Madison, WI, USA) at 47 °C for one hour. Peptides were eluted with 40 µL of 50 mM TEAB followed by 40 µL of 0.2% aqueous formic acid and by 35 µL of 50% acetonitrile (ACN) containing 0.2% formic acid. Peptides were, finally, vacuum-dried down.

### 4.10. nanoLC-MS/MS Measurements

Samples were resuspended in 100 µL of 10% ACN, 0.1% TFA in HPLC-grade water. For each run, 1 µL was injected in a nanoRSLC-Q Exactive PLUS (RSLC Ultimate 3000) (Thermo Scientific, Waltham, MA, USA). Peptides were loaded onto a µ-precolumn (Acclaim PepMap 100 C18, cartridge, 300 µm id × 5 mm, 5 µm) (Thermo Scientific), and were separated on a 50 cm reversed-phase liquid chromatographic column (0.075 mm ID, Acclaim PepMap 100, C18, 2 µm) (Thermo Scientific). Chromatography solvents were (A) 0.1% formic acid in water, and (B) 80% ACN, 0.08% formic acid. Peptides were eluted from the column with the following gradient, 5% to 40% B (180 min), 40% to 80% (1 min). At 181 min, the gradient stayed at 80% for 5 min and, at 187 min, it returned to 5% to re-equilibrate the column for 20 min before the next injection. One blank was run between each series to prevent sample carryover. Peptides eluting from the column were analyzed by data dependent MS/MS, using a top-10 acquisition method. Peptides were fragmented using a higher-energy collisional dissociation (HCD). Briefly, the instrument settings were as follows: Resolution was set to 70,000 for MS scans and 17,500 for the data dependent MS/MS scans in order to increase speed. The MS AGC target was set to 3 × 10^6^ counts with a maximum injection time set to 60 ms, while the MS/MS AGC target was set to 1 × 10^5^ with a maximum injection time set to 60 ms. The MS scan range was from 400 to 2000 m/z. Dynamic exclusion was set to a 30 s duration. We analyzed four independent biological replicates per condition, each in technical triplicates.

### 4.11. Data Processing Following LC-MS/MS Acquisition

The MS files were processed with the MaxQuant software version 1.5.8.3 (MPI of Biochemistry, Martinsried, Germany) and searched with Andromeda search engine against the database of *Homo sapiens* from Swiss-Prot 07/2017. To search parent mass and fragment ions, we set an initial mass deviation of 4.5 and 20 ppm, respectively. The minimum peptide length was set to seven amino acids and a strict specificity for trypsin cleavage was required, allowing up to two missed cleavage sites. Carbamidomethylation (Cys) was set as a fixed modification, whereas oxidation (Met) and N-term acetylation were set as variable modifications. The false discovery rates (FDRs) at the protein and peptide level were set to 1%. Scores were calculated in MaxQuant as described previously [54]. The reverse and common contaminants hits were removed from the MaxQuant output. Proteins were quantified according to the MaxQuant label-free algorithm using LFQ (Label-free Quantification) intensities [54,55]. A match between runs was allowed.

The proteomes of MUT-KO and WT (used as a control) were analyzed with the Perseus software version 1.6.0.7 (MPI of Biochemistry, Martinsried, Germany), freely available at www.perseus-framework.org. LFQ data were log2-transformed, and proteins that were identified in all four replicates of the WT or MUT-KO were retained for statistical analysis. Missing values were replaced by creating a Gaussian distribution of random numbers with a standard deviation of 30% relative to the standard deviation of the measured values and three standard deviation downshifts of the mean. The log2 protein difference of the intensities was then calculated between the two analyzed groups in order to describe the variation in protein abundance. Data were then analyzed by a *t*-test (FDR = 1%, S0 = 0.5) and represented on a volcano plot, after imputation of the missing values. The principal component analysis (PCA) plot and the heatmap were obtained using MetaboAnalyst 4.0 tool (Wishart Research Group, University of Alberta, Edmonton, AB, Canada) [56].

The mass spectrometry proteomics data have been deposited to the ProteomeXchange Consortium (available at http://www.proteomexchange.org) via the PRIDE partner repository with the dataset identifier PXD017977 [57].

### 4.12. Bioinformatic Analyses

A bioinformatic analysis was carried out using the EnrichR software (Ma’ayan Laboratory, Mount Sinai Center for Bioinformatics, New York, NY, USA) [58]. Down- and upregulated proteins from the proteomic dataset were analyzed separately. Biological process, molecular function, and cellular component GO terms for both datasets were extracted according to the *p*-value ranking. Within the EnrichR software, the global regulated dataset was analyzed with the Human Phenotype Ontology tool [59], in order to retrieve typical phenotypic traits related to MMA.

The STRING (Search Tool for the Retrieval of Interacting Genes) online version 11 (STRING CONSORTIUM 2020) [60,61] was used for the generation of the interacting network of mitochondrial proteins. A minimum of the required interaction score of 0.150 (low confidence) was set. From STRING, the most statistically significant and non-redundant biological processes in which the selected mitochondrial proteins resulted involved were retrieved.

## 5. Conclusions

Actually, our results describe the proteomic landscape of a MUT-KO cell model in the view of shedding light on altered cellular pathways in MMA. Here, we propose a link between MMA and molecular alterations involving modification in cellular architecture and morphology, metal homeostasis impairment, increased susceptibility to stress, and mitochondrial dysfunctionality. The overall alterations contribute in concert into provoking cellular and tissue damage in patients. Several protein alterations described in the cellular model can be, in fact, strictly related to clinical features characterizing MMA patients.

## Figures and Tables

**Table 1:** Gene ontology (GO) terms enrichment from the bioinformatic analysis of dysregulated proteome in MUT-KO.

GO Term	Downregulated	Upregulated
BP	Zinc II ion transmembrane transport (GO:0071577)	Intermediate filament bundle assembly (GO:0045110)
BP	Positive regulation of cell communication (GO:0010647)	Coenzyme biosynthetic process (GO:0009108)
MF	Divalent inorganic cation transmembrane transporter activity (GO:0072509)	Oxidoreductase activity, acting on the CH-OH group of donors, NAD or NADP as acceptor (GO:0016616)
CC	Autophagosome (GO:0005776)	Mitochondrion (GO:0005739)
CC	Autolysosome (GO:0044754)	Cytoskeleton (GO:0005856)

BP: Biological process; MF: Molecular function; CC: Cellular component.

**Table 2:** Subset of dysregulated mitochondrial proteins identified by LFQ proteomics in MUT-KO.

UniProt Accession	Protein Name	Gene Name	Regulation	Log2 Difference
P22033	Methylmalonyl-CoA mutase, mitochondrial	*MUT*	DOWN	−8.4
P23434	Glycine cleavage system H protein, mitochondrial	*GCSH*	DOWN	−3.8
Q99595	Mitochondrial import inner membrane translocase subunit Tim17-A	*TIMM17A*	DOWN	−3.3
Q8N4T8	Carbonyl reductase family member 4	*CBR4*	DOWN	−3.1
Q9NUQ2	1-acyl-sn-glycerol-3-phosphate acyltransferase epsilon	*AGPAT5*	DOWN	−3.0
Q9Y375	Complex I intermediate-associated protein 30, mitochondrial	*NDUFAF1*	DOWN	−2.7
Q13772	Nuclear receptor coactivator 4	*NCOA4*	DOWN	−1.1
P02794	Ferritin heavy chain	*FTH1*	DOWN	−0.9
P32119	Peroxiredoxin-2	*PRDX2*	UP	0.8
P27105	Erythrocyte band 7 integral membrane protein	*STOM*	UP	0.8
Q13057	Bifunctional coenzyme A synthase	*COASY*	UP	0.9
Q9H2U2	Inorganic pyrophosphatase 2, mitochondrial	*PPA2*	UP	0.9
Q6YN16	Hydroxysteroid dehydrogenase-like protein 2	*HSDL2*	UP	1.0
Q16698	2,4-dienoyl-CoA reductase, mitochondrial	*DECR1*	UP	1.0
P35270	Sepiapterin reductase	*SPR*	UP	1.0
P23786	Carnitine O-palmitoyltransferase 2, mitochondrial	*CPT2*	UP	1.0
Q8WVC6	Dephospho-CoA kinase domain-containing protein	*DCAKD*	UP	1.0
P35914	Hydroxymethylglutaryl-CoA lyase, mitochondrial	*HMGCL*	UP	1.0
P12532	Creatine kinase U-type, mitochondrial	*CKMT1A*	UP	1.1
Q9BRX8	Redox-regulatory protein FAM213A	*FAM213A*	UP	1.2
Q9NP58	ATP-binding cassette sub-family B member 6, mitochondrial	*ABCB6*	UP	1.4
Q9NRG7	Epimerase family protein SDR39U1	*SDR39U1*	UP	1.4
Q9Y2Z9	Ubiquinone biosynthesis monooxygenase COQ6, mitochondrial	*COQ6*	UP	1.7
Q14790	Caspase-8	*CASP8*	UP	1.8
Q86UT6	NLR family member X1	*NLRX1*	UP	2.1
Q9BVL4	Selenoprotein O	*SELO*	UP	2.4
Q6PML9	Zinc transporter 9	*SLC30A9*	UP	2.4
O95139	NADH dehydrogenase [ubiquinone] 1 beta subcomplex subunit 6	*NDUFB6*	UP	2.7
A8MXV4	Nucleoside diphosphate-linked moiety X motif 19, mitochondrial	*NUDT19*	UP	3.6
Q8IYQ7	Threonine synthase-like 1	*THNSL1*	UP	3.7
Q9BSF4	Uncharacterized protein C19orf52	*C19orf52*	UP	4.0
Q9P0M9	39S ribosomal protein L27, mitochondrial	*MRPL27*	UP	4.5
P00395	Cytochrome c oxidase subunit 1	*COX1*	UP	5.7

**Table 3:** Dysregulated proteins in MUT-KO involved in cell architecture modification.

UniProt Accession	Protein Name	Gene Name	Regulation	Log2 Difference	Cell Localization
Q93063	Exostosin-2	*EXT2*	DOWN	−6.0	ER, ERS, GA
O00622	Protein CYR61	*CYR61*	DOWN	−5.9	ER, ERS
Q9P2M7	Cingulin	*CGN*	DOWN	−3.1	CK, PM
Q2PZI1	Probable C-mannosyltransferase DPY19L1 Alpha-1,2-mannosyltransferase ALG9 Fibronectin Mannosyl-oligosaccharide 1,2-alpha-mannosidase IA Annexin A3 Follistatin-related protein 1	*DPY19L1*	DOWN	−2.9	N
Q9H6U8		*ALG9*	DOWN	−2.8	ER
P02751		*FN1*	DOWN	−2.7	ER, ERS, PM
P33908		*MAN1A1*	DOWN	−2.7	C, ER, ERS, GA
P12429		*ANXA3*	DOWN	−2.6	C, ERS, PM
Q12841		*FSTL1*	DOWN	−2.5	ER, ERS
Q12929	Epidermal growth factor receptor kinase substrate 8	*EPS8*	DOWN	−2.1	C, ERS, PM
P08670	Vimentin	*VIM*	DOWN	−2.0	C, CK, ERS, N, P, PM
P13591	Neural cell adhesion molecule 1	*NCAM1*	DOWN	−1.6	C, ERS, GA, PM
P34741	Syndecan-2	*SDC2*	DOWN	−0.9	ER, ERS, GA, L, PM
Q16706	Alpha-mannosidase 2	*MAN2A1*	DOWN	−0.9	ERS, GA
P23352	Anosmin-1	*KAL1*	DOWN	−0.8	ERS, PM
P10253	Lysosomal alpha-glucosidase	*GAA*	UP	+0.8	ERS, L, PM
P12830	Cadherin-1	*CDH1*	UP	+3.9	CK, E, ERS, GA, PM
Q6Y288	Beta-1,3-glucosyltransferase	*B3GALTL*	UP	+5.8	ER

C: Cytosol; CK: Cytoskeleton; E: Endosome; ER: Endoplasmic reticulum; ERS: Extracellular region or secreted; GA: Golgi apparatus; L: Lysosome; N: Nucleus; P: Peroxisome; PM: Plasma membrane.

**Table 4:** Dysregulated proteins in MUT-KO involved in metal homeostasis.

UniProt Accession	Protein Name	Gene Name	Regulation	Log2 Difference	Cell Localization
Q9ULF5	Zinc transporter ZIP10	*SLC39A10*	DOWN	−4.5	PM
Q8IUH5	Palmitoyltransferase ZDHHC17	*ZDHHC17*	DOWN	−4.2	GA, PM
Q13433	Zinc transporter ZIP6	*SLC39A6*	DOWN	−2.6	ER, PM
Q9NY26	Zinc transporter ZIP1	*SLC39A1*	DOWN	−1.1	ER, PM
Q13772	Nuclear receptor coactivator 4	*NCOA4*	DOWN	−1.1	L, N
P02794	Ferritin heavy chain	*FTH1*	DOWN	−0.9	C, ERS, L, N
P48200	Iron-responsive element-binding protein 2	*IREB2*	UP	+0.9	C, M
Q9NP58	ATP-binding cassette sub-family B member 6, mitochondrial	*ABCB6*	UP	+1.4	C, E, ER, ERS, GA, M
Q6PML9	Zinc transporter 9	*SLC30A9*	UP	+2.4	CK, ER, N

C: Cytosol; CK: Cytoskeleton; E: Endosome; ER: Endoplasmic reticulum; ERS: Extracellular region or secreted; GA: Golgi apparatus; L: Lysosome; M: Mitochondrion; N: Nucleus; PM: Plasma membrane.

**Table 5:** Dysregulated proteins in MUT-KO involved in mitochondrial alterations.

UniProt Accession	Protein Name	Gene Name	Regulation	Log2 Difference	Cell Localization
P23434	Glycine cleavage system H protein, mitochondrial	*GCSH*	DOWN	−3.8	M
P35914	Hydroxymethylglutaryl-CoA lyase, mitochondrial	*HMGCL*	UP	+0.9	C, M, P
Q13057	Bifunctional coenzyme A synthase	*COASY*	UP	+0.9	ERS, M
Q16698	2,4-dienoyl-CoA reductase, mitochondrial	*DECR1*	UP	+1.0	C, M, N
P23786	Carnitine O-palmitoyltransferase 2, mitochondrial	*CPT2*	UP	+1.0	M, N
Q6P1M0 A8MXV4	Long-chain fatty acid transport protein 4 Nucleoside diphosphate-linked moiety X motif 19, mitochondrial	*SLC27A4* *NUDT19*	UP UP	+1.1 +3.6	ER, PM C, P

C: Cytosol; ER: Endoplasmic reticulum; ERS: Extracellular Region or Secreted; M: Mitochondrion; N: Nucleus; P: Peroxisome; PM: Plasma Membrane.

## Supplementary Materials

The following are available online at https://www.mdpi.com/1422-0067/21/14/4998/s1; Supplementary Figure S1: Analysis of MUT mRNA levels by qRT-PCR in WT, MUT-KO, and MUT-Rescue cells; Supplementary Figure S2: Western blot detection of MUT protein in the samples used for proteomic analysis; Supplementary Figure S3: GO term enrichment for the regulated proteome of MUT-KO cells by EnrichR; Supplementary Table S1: Primers sequences used for qRT-PCR analyses; Supplementary Table S2: Gene Ontology enrichment analysis of the proteomic dataset by EnrichR.
