# Supplementary material for: Proteomics Reveals that Methylmalonyl-CoA Mutase Modulates Cell Architecture and Increases Susceptibility to Stress
Source: Int J Mol Sci. 2020 Jul 15;21(14):4998. doi: 10.3390/ijms21144998 (PMC7403994; doi:10.3390/ijms21144998)
Supplement: Supplementary file 1 [file ijms-21-04998-s001.zip › supplementary files/Supplementary Figures.docx]

Proteomics reveals that methylmalonyl-CoA mutase modulates cell architecture and increases susceptibility to stress

Michele Costanzo ^1,2,§^, Marianna Caterino ^1,2,§^, Armando Cevenini ^1,2^, Vincent Jung ^3^, Cerina Chhuon ^3^, Joanna Lipecka ^3^, Roberta Fedele ^2^, Ida Chiara Guerrera ^3,*^ and Margherita Ruoppolo ^1,2,*^

^1^ Department of Molecular Medicine and Medical Biotechnology, School of Medicine, University of Naples Federico II, Naples, Italy; [michele.costanzo@unina.it](mailto:michele.costanzo@unina.it) (M.C.), [marianna.caterino@unina.it](mailto:marianna.caterino@unina.it) (M.C.), [armando.cevenini@unina.it](mailto:armando.cevenini@unina.it) (A.C.), [margherita.ruoppolo@unina.it](mailto:margherita.ruoppolo@unina.it) (M.R.)

^2^ CEINGE - Biotecnologie Avanzate s.c.ar.l., Naples, Italy; [fedeler@ceinge.unina.it](mailto:fedeler@ceinge.unina.it) (R.F.)

^3^ Proteomics Platform Necker, Université de Paris - Structure Fédérative de Recherche Necker, Inserm US24/CNRS UMS3633, Paris, France; [vincent.jung@inserm.fr](mailto:vincent.jung@inserm.fr) (V.J.), [cerina.chhuon@inserm.fr](mailto:cerina.chhuon@inserm.fr) (C.C.), [joanna.lipecka@inserm.fr](mailto:joanna.lipecka@inserm.fr) (J.L.), [chiara.guerrera@inserm.fr](mailto:chiara.guerrera@inserm.fr) (I.C.G)

***** Co-correspondence: [margherita.ruoppolo@unina.it](mailto:margherita.ruoppolo@unina.it); Tel.: 39-081-3737850 (M.R.) and [chiara.guerrera@inserm.fr](mailto:chiara.guerrera@inserm.fr) (I.C.G.)

^§^ These authors equally contributed to this work.

**Supplementary Figures**


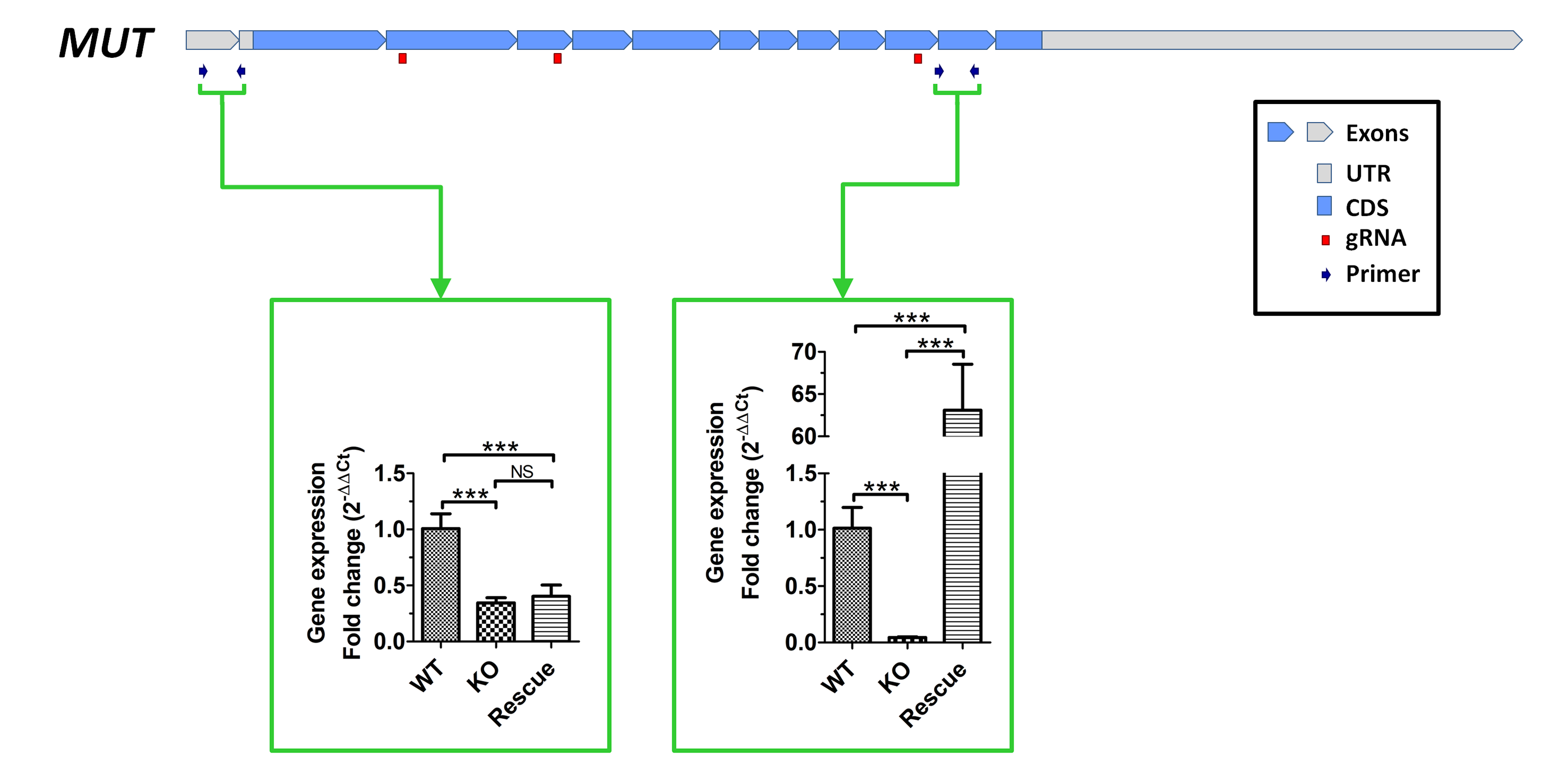


**Supplementary Figure S1. qRT-PCR analysis of MUT mRNA levels in WT, MUT-KO and MUT-Rescue cells.** The real-time PCR was performed with two set of specific primers: MUT primer pair 1 (MPP1): Forward 5’-TTTGTGTGGGCTTGGTGAGG-3’, Reverse 5’-AAATAAGAACTGACTGGTAGGC-3’; MUT primer pair 2 (MPP2): Forward 5’-CCTCTTTTCCAGACTCCTCG -3’, Reverse 5’- TTAAGTTCTTTGATGAGTTCAGG -3’. Three independent experiments were performed for each sample (average values and standard deviation of triplicate determinations are shown). Relative gene expression was normalized to β-actin, α-tubulin and RNA polymerase II genes and determined using the 2^-ΔΔCt^ method. Statistical significance was calculated by one-way two tail paired *t*-test. p-value are indicated as follows: *=p<0.05; **=p<0.01, *** p =<0.005.

MPP1 matches upstream with respect to regions targeted by the guide RNAs (gRNAs) of the CRISPR/CAS9 kit, while MPP2 matches downstream with respect to CRISPR-targeted zones. Analysis results indicate that in MUT-KO cells the transcription of the upstream region (MPP1) of *MUT* gene (likely containing promoting and regulatory CIS-elements for transcription) is hardly compromised, while transcripts containing the downstream region (MPP2) are almost absent. With regard to MUT-Rescue cells [which are derived from MUT-KO ones by stable transfection of a plasmidic construct containing MUT cDNA coding sequence (CDS)], the results with MMP1 are obviously comparable to those in MUT-KO, since MMP1 amplify a zone in the 5’-UTR which is not contained in the MUT CDS. On the other hand, MPP2 matches a sequence that is contained in the Rescue construct which is regulated by a strong constitutive promoter element (Cytomegalovirus promoter) and thus the amplification with this last primer pair gives expression values (after normalizations) that are more than 60-fold higher than those obtained in WT cells.


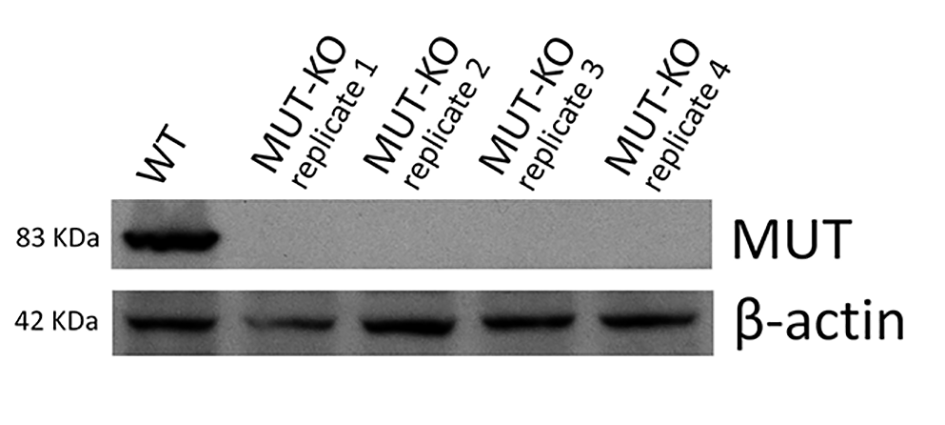


**Supplementary Figure S2.** **Western blot detection of MUT protein in the samples used for proteomic analysis.** WT and MUT-KO samples (4 biological replicates per condition) were tested by WB for the detection of MUT protein prior proteomic analysis. MUT was detected only in the WT lane and not in the MUT-KO samples. In this WB, only one WT sample was used as representative for all four WT replicates. β-actin was used as loading control.


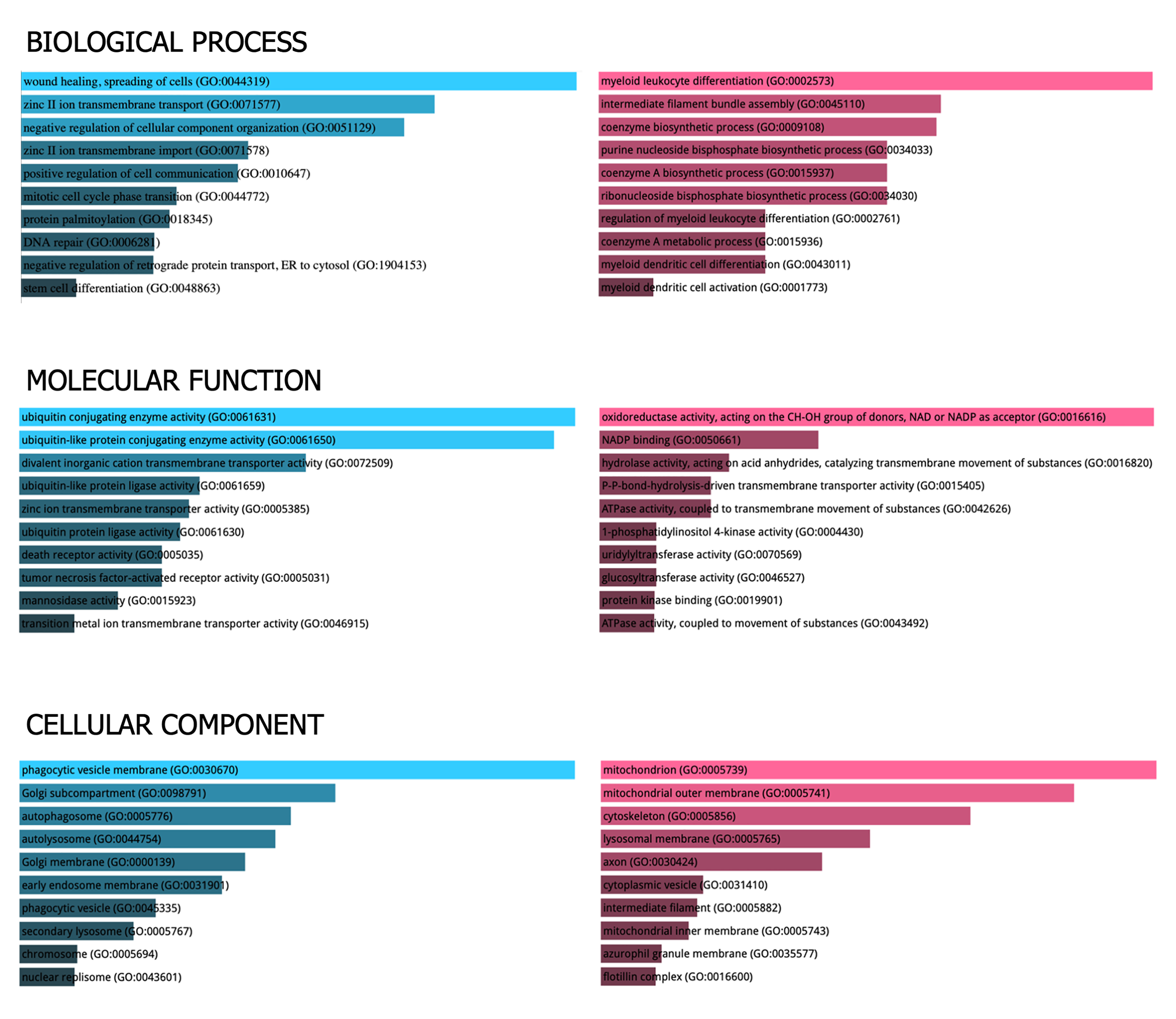


**Supplementary Figure S3.** **GO term enrichment for the regulated proteome of MUT-KO cells by EnrichR.** Down- and up-regulated proteins were clustered separately (blue and pink bars, respectively) into Biological Process, Molecular Function and Cellular Component GO terms using EnrichR software. GO terms per category are ordered according to decreasing p-value ranking significance.
